# A case report: Infrazygomatic approach neurocryoablation of the sphenopalatine ganglion under cone beam computer tomography guidance in treating refractory headaches

**DOI:** 10.1016/j.inpm.2025.100706

**Published:** 2025-11-25

**Authors:** Logan F. Condon, Ryu Komatsu, Jiang Wu

**Affiliations:** a University of Washington Medical Scientist Training Program, Seattle, WA, USA; b University of Washington School of Medicine, Seattle, WA, USA; c University of Washington Department of Anesthesiology & Pain Medicine, Seattle, WA, USA; d Multi-Specialty Anesthesiology, Integrated Hospital-Care Institute, Cleveland Clinic, Cleveland, OH, USA

**Keywords:** Trigeminal autonomic cephalalgia, Sphenopalatine ganglion, Neurocryoablation, Cone beam CT guidance

## Abstract

**Introduction:**

The sphenopalatine ganglion (SPG) drives the cranial autonomic symptoms associated with trigeminal autonomic cephalalgias, migraine, and trigeminal neuralgia. Infrazygomatic approach SPG block serves as the gold standard for both prognostic and therapeutic intervention for management of refractory headache with autonomic symptoms. However, this intervention often provides only short-term relief. Identification of novel interventions that provide more durable relief is imperative.

**Case:**

An 84-year-old female with chronic headache featuring autonomic cranial symptoms that failed both medication and conservative intervention management. Patient responded well to infrazygomatic approach SPG block using combined cone beam CT and fluoroscopy guidance, however, experienced fading therapeutic response over the years. Subsequently, patient underwent neurocryoablation of the SPG, from which, satisfactory and sustained improvement of her headache pain was achieved at six month follow up.

**Conclusion:**

This is the first case demonstrating the clinical feasibility and therapeutic outcomes of SPG neurocryoablation in treating refractory headaches.

## 1 Introduction

Trigeminal autonomic cephalalgias (TACs) are primary headache disorders with debilitating unilateral trigeminal distribution pain with associated ipsilateral cranial autonomic symptoms. The attacks are episodic, generally lasting less than 4 h, and occur in clusters, where attacks occur up to multiple times daily for weeks to months [1,2]. Autonomic symptoms of TACs include lacrimation, eyelid edema, conjunctival injection, facial sweating, ptosis, and rhinorrhea. These symptoms are believed to be the result of aberrant parasympathetic outflow from the sphenopalatine ganglion (SPG) [1,2]. The SPG is a parasympathetic ganglion, however it also acts as a conduit for post-ganglionic sympathetic and sensory fibers innervating cranial structures [3]. The SPG resides in the pterygopalatine fossa (PPF) and is anatomically proximal to the ophthalmic and maxillary branches of the trigeminal nerve (CN V), with maxillary branch CN V sensory fibers running through the SPG [3]. CN V mediates the pain sensation associated with TACs [2]. Collectively, SPG and CN V fibers account for the sensory and autonomic symptoms of TACs. Given the anatomic proximity and intermingling of these two structures, interventional techniques targeting the SPG and proximate CN V fibers are important in the treatment of medically refractory TACs [4,5].

SPG blockade has been effectively used to treat TACs for more than 100 years [5,6]. Transnasal, transoral, and transfacial approaches have been employed. However, the percutaneous infrazygomatic SPG block is the most direct approach, and as such is the most commonly employed [7]. Despite the high abortive success, only the short-term pain relief is provided by local anesthetics [5]. Therefore, Radiofrequency ablation (RFA) techniques, both thermal and low temperature pulsed, have been utilized to obtain the therapeutic benefit [5,8]. However, neurocryoablation has been most widely described in the treatment of post-thoracotomy pain through intercostal nerve cryoablation. This clinical experience has supported its extension to other pain syndromes [[9], [10], [11]]. To explore an alternative to RFA and to provide more durable relief than local anesthetic, we employed neurocryoablation of the SPG. This case report details the novel and successful application of infrazygomatic neurocryoablation of the SPG with Cone Beam CT (CBCT) guidance for the treatment of TACs.

## 2 Case report

### 2.1 History

An 84-year-old female with medical history of intractable chronic migraines, paroxysmal hemicrania, cervicalgia, and chronic sinusitis status post bilateral uncinectomies and partial internal ethmoidectomies, who presented for progressively worsening headaches over 5-years. She had recurrent episodes of stabbing pain located primarily in the left side of the face deep to the base of nose lasting between 8 and 12 minutes. The pain was 10/10 intensity and caused significant lacrimation and nasal discharge. Pain episodes occurred throughout the day but most frequently were in the evening. Her physical exam was normal without focal findings. Maxillo-facial CT without contrast revealed postsurgical alterations with focal area of mucosal thickening/opacification noted along the right greater than left anterior ethmoids and without signs suggesting any acute sinusitis. She had previously failed treatment with Topiramate, Indomethacin, Verapamil, Gabapentin, Nortriptyline, Valproate, Oxcarbazepine, Duloxetine, and external vagal nerve stimulator. Her presentation was most consistent with paroxysmal hemicrania based on the duration of the episodes, however, does not meet criteria due to the lack of response to Indomethacin [1]. She received multiple percutaneous infrazygomatic SPG blocks with 2 ml mixture of 0.5 % bupivacaine (1 ml) and 40 mg triamcinolone (1 ml). The effect of repeated block was initially satisfactory, with complete relief lasting 2 weeks and moderate relief lasting 3–4 weeks, but therapeutic pain benefits gradually faded over time. After the final block procedure, she felt no relief from her pain. Based on her previously positive response to the blocks, after full disclosure of risk/benefit/alternative, she underwent left-sided infrazygomatic approach sphenopalatine ganglion cryoablation with Cone-beam CT guidance.

### 2.2 Patient monitoring and position

This procedure was performed conforming with standard ASA monitoring under IV sedation. The patient was positioned supine with her head, neck, and chest supported in a neutral position. The patient was cooperative with the positioning process. The angles of the mandible were adjusted so the pterygopalatine fosses were clearly superimposed on each other under the lateral fluoroscopic view. The left-cheek area surrounding the zygomatic arch was prepared with chlorhexidine and draped in a sterile fashion. Sterile technique was maintained throughout the procedure.

### 2.3 3D CT reconstruction and planning of needle pathway

**Fig. 1:**
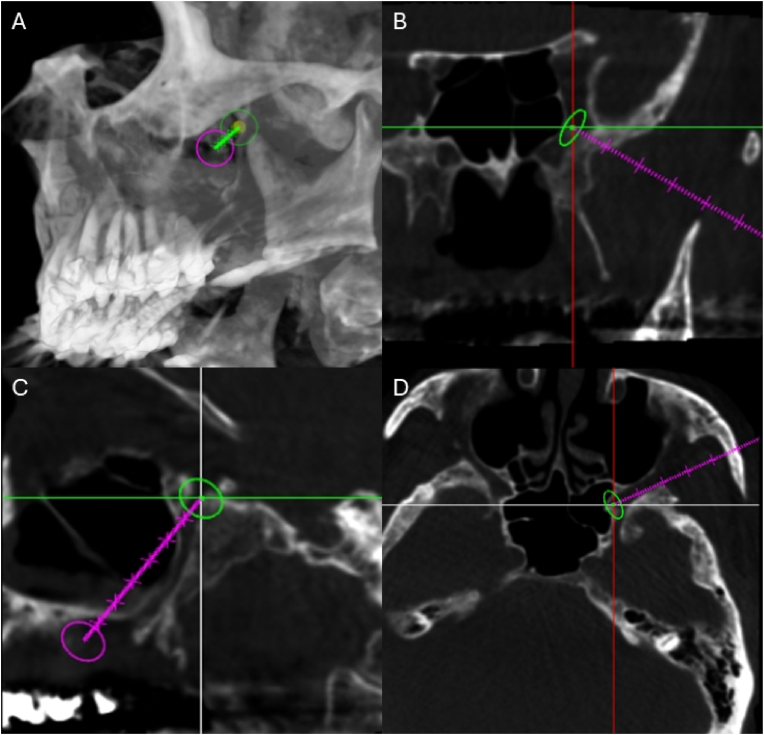
A) 3-D reconstruction of cone beam CT image used to determine safe trajectory for cryoablation probe. Green line: probe trajectory, green circle: probe tip, and red circle: probe tail. B) Coronal CT image demonstrating probe trajectory inferior to zygomatic arch with probe tip overlying pterygopalatine fossa. C) Sagittal CT image demonstrating probe tip overlying pterygopalatine fossa. D) Horizontal CT image demonstrating probe trajectory through pterygomaxillary fissure with probe tip overlying pterygopalatine fossa. B-D) Purple line: probe trajectory, green circle: probe tip. (For interpretation of the references to colour in this figure legend, the reader is referred to the Web version of this article.)

An 8-s 3-dimensional (3-D) CT reconstruction of the maxillo-facial area was obtained via a single C-arm rotation scan using a Phillip Allura Systems (Cone Beam CT). Phillip Allura Systems 3D rendering and editing software was used to determine an optimal needle trajectory into the PPF that avoided bony structures. This was accomplished prior to the procedure by using the Entry and Progress View function to toggle between coronal and horizontal views of the patient's cranial anatomy while tracing a needle trajectory beginning under the zygomatic arch and traversing the pterygomaxillary fissure. Safe trajectory of the entire cryoablation probe was planned on 3-D reconstruction of CBCT image (Fig. 1 A). The probe was mapped inferior to the zygomatic arch ending within the PPF on the coronal plane (Fig. 1 B), within the PPF on sagittal plan (Fig. 1C), and through pterygomaxillary fissure ending within the PPF on the horizontal plan (Fig. 1 D).

### 2.4 Entry view and progress view of angiocath placement

**Fig. 2:**
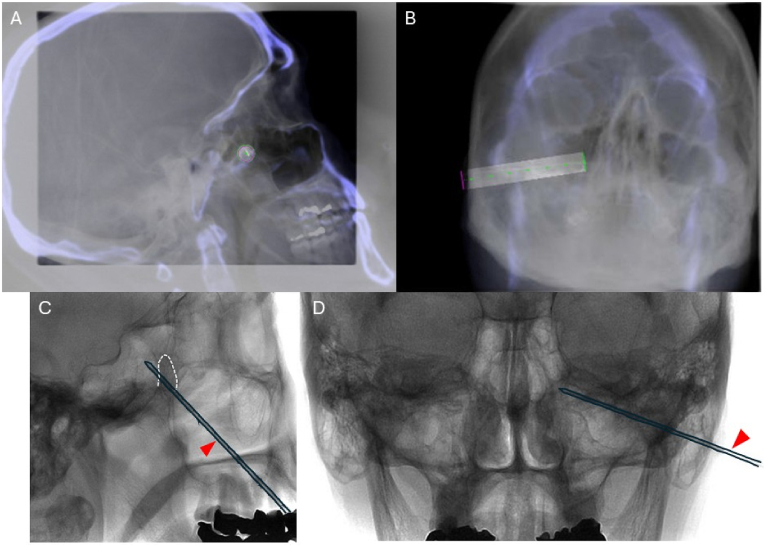
A) In the entry view of the angiocath, a 2D fluoroscopy image is superimposed on the reconstructed cone beam–based CT (CBCT) imaging, depicting the angiocath being advanced coaxially along the planned trajectory toward the pterygopalatine fossa. The tail of the angiocath is in overlap with the tip of the needle, so the angiocath axis appears as a single overlapping green/pink circle on the background 3-D CBCT imaging. B) In the progress view, the shaft of angiocath is in overlap with the planned trajectory in green line and the tip of angiocath stopped at the target pterygopalatine fossa. C) In lateral view, the final position of angiocath was confirmed with tip within pterygopalatine fossa. White dashed outline: pterygopalatine fossa, red triangle: 14g 3 1/2″ blunt angiocath. D) In posterior-anterior view, the final position of angiocath was confirmed with tip at the level of middle turbinate of lateral wall of nasal cavity. (For interpretation of the references to colour in this figure legend, the reader is referred to the Web version of this article.)

First, local anesthesia was applied at the skin entry site. A 2D fluoroscopy image is superimposed on the reconstructed CBCT scan. In the entry view, the 14 GAUGE 3 1/2″ angiocath (Trocar) with the sharp stylet was advanced coaxially along the preplanned trajectory toward the pterygopalatine fossa. The alignment of the angiocath tail and tip creates a single overlapping marker on the CBCT background, confirming trajectory accuracy (Fig. 2 A). In the progress view, the angiocath tip is verified reaching the predetermined target point after advancement along the designed pathway under intermittent fluoroscopic guidance (Fig. 2 B). The integration of CBCT-based planning with real-time fluoroscopic monitoring is highlighted to ensure precise needle placement.

### 2.5 Lateral view and posterior-anterior view of final angiocath position

In lateral view of skull, the final angiocath position is confirmed in a medial, cephalad, and slightly anterior direction with the tip within pterygopalatine fossa (Fig. 2C). In posterior-anterior view, the final position of angiocath was confirmed with the tip of angiocath in line with the lateral wall of the nasal cavity, at the level of the middle turbinate (Fig. 2 D). The trocar tip was intentionally placed slightly anterior to the PPF in an attempt to target both the SPG and fibers originating from the maxillary branch of the trigeminal nerve.

### 2.6 Neurocryoablation of SPG

The stylet was removed and the 17 GAUGE neurocryoabalation probe was inserted with the 1 cm active tip exposed within the PPF. Then 1ml of 0.5 % bupivicaine was injected for local anesthesia. Four cycles of lesioning were carried out at −100 °C for 180 seconds following each repositioning. After lesioning, 1 mL of bupivacaine 0.5 % and 40 mg of triamcinolone was injected with the aim of preventing post-procedure neuritis. The total procedure time was less than 30 minutes. No adverse effects were noted. Post-procedure the patient was transferred to the recovery area in stable condition. She noted initial pain relief. She reported numbness of upper soft palate, a result of local anesthetic, which suggested placement of the probe near the SPG.

### 2.7 Patient outcomes

The first cryoablation provided approximately 50 % improvement, but the patient continued to experience persistent headache symptoms. After an extensive discussion, the patient requested a repeat procedure, hoping to achieve cumulative benefits. The second cryoablation further increased pain relief to about 75 % at two-week follow-up. She described complete resolution of the sharp and stabbing pain, lacrimation and nasal discharge that had previously accompanied her headaches. She continued to experience low-grade dull headache pain but stated she was happy with the procedure overall. This level of relief was persistent at the two-month follow-up appointment. In addition to the pain relief observed after SPG ablation, the patient also reported a notable improvement in her ability to sleep. At the six-month follow-up the patient continued to experience relief from her sharp and stabbing left-sided maxillary pain, however she did report headache pain affecting her forehead.

## 3 Discussion

The SPG is a well-established interventional target for TACs. The SPG is hidden inside of PPF a small inverted pyramidal fossa located between the maxillary bone anteriorly, the pterygoid process posteriorly, and orbital apex superiorly [3]. In adults, the pterygopalatine fossa (PPF) typically measures about 20–22 mm in height (superior–inferior) and has an estimated volume of approximately 1 cm^3^, although values vary depending on how its boundaries are defined. Variations in the orientation and size of the pterygomaxillary fissure make access to the SPG within the PPF technically challenging. To date, SPG ablation has predominantly been performed under 2-D fluoroscopic guidance using RFA [5,8]. The RFA needle is usually a 20- or 21-gauge curved-tip cannula, which facilitates maneuverability with the “walking technique” under fluoroscopy. The target endpoint is reached when the needle tip aligns with the lateral wall of the nasal cavity at the level of the middle turbinate, as confirmed in the anteroposterior (AP) view. By contrast, during neurocryoablation of SPG, infrazygomatic placement using a 14-gauge straight angiocath with sharp stylet into the PPF requires advancing below the zygomatic arch and traversing the narrow pterygomaxillary fissure, demanding a predetermined and straight pathway to avoid bony structures. 3-D CBCT image guidance enables accurate pre-procedural identification of both the optimal skin entry site and a precise, straight probe trajectory. This predetermined pathway then guides the subsequent use of a coaxial needle insertion technique under standard 2-D fluoroscopic guidance. For these reasons, 3-D image guidance likely enhances both the safety and effectiveness of neurocryoablation procedures with a much larger trocar while maintaining patient comfort.

Conventional thermal RFA produces focal tissue heating that results in coagulation necrosis followed by fibrous scar formation. Histological and imaging studies across cardiac and non-cardiac applications consistently demonstrate that scar formation is both universal and intended, with an incidence approaching 100 % in the ablated zone. Nevertheless, neurocryoablation disrupts the nerve structure and creates Wallerian degeneration while leaving the myelin sheath and endoneurium intact [10,11]. Therefore, neurocryoablation provides cyroanalgesia while maintaining nerve structure partially intact [10,11]. As a result of the difference in their mechanism of actions, neurocryoablation has theoretical advantages in potentially reducing scar formation, neuritis and neuropathic pain. Furthermore, a 17-gauge neuroablation probe with a 1-cm active tip produces an ice ball approximately 1 cm in diameter around the probe tip. Performing multiple freeze–thaw cycles with needle repositioning can generate a larger ice matrix, thereby expanding the effective area of neurolysis inside of a small PPF [10]. Neurocryoablation may therefore represent a preferable option when repeated neuroablative treatments are anticipated.

Stationary Computer Tomography (CT) guidance is not typically available to the pain interventionist due to unfavorable cost-efficiency ratios and concerns of excessive radiation exposure [12]. However, fluoroscopy only provides 2-D imaging guidance, significantly increasing the difficulty of determining optimal needle trajectory to the FFP. Cone beam CT (CBCT) uniquely provides the function of both CT and fluoroscopy using a C-arm machine. It allows precise 3-D needle planning and 2-D needle guidance, while ensuring tight needle control and detailed monitoring of contrast spread. CBCT guidance provides more geometric accuracy and higher spatial resolution of structures in the head and neck and allows for more accurate and safer trajectory planification and placement of the cryoablation probe [13].

This case report demonstrates the use of CBCT guided ablation of pterygopalatine fossa structures, resulting in clinically significant improvement of trigeminal autonomic cephalalgia symptoms. This case highlights a novel approach for the management of treatment refractory TACs.

## Declarations

No funding was received for this case study. The authors do not have any financial interests associated with the technique reported above. The authors do not have any competing interests to disclose. Prior to preparing this case report the authors obtained informed consent from the patient to participate and publish. All data associated with this study are included in the published case report. All authors contributed to the case report's content. The procedure described in this case report was performed by Dr. Jiang Wu. The manuscript was written by Dr. Jiang Wu, MD and Logan Condon, PhD. All authors commented on and revised the drafts of this manuscript. All authors read and approved the final manuscript.

## Declaration of competing interest

The authors declare that they have no known competing financial interests or personal relationships that could have appeared to influence the work reported in this paper.
